# Effect of heartworm disease and heartworm-associated respiratory disease (HARD) on the right ventricle of cats

**DOI:** 10.1186/s13071-017-2451-7

**Published:** 2017-11-09

**Authors:** Randolph L. Winter, A. Ray Dillon, Russell C. Cattley, Byron L. Blagburn, D. Michael Tillson, Calvin M. Johnson, William R. Brawner, Elizabeth G. Welles, Sharon Barney

**Affiliations:** 1 Department of Clinical Sciences, College of Veterinary Medicine, Auburn University, Auburn, AL 36849 USA; 2 Department of Pathobiology, College of Veterinary Medicine, Auburn University, Auburn, AL USA

**Keywords:** Feline, Myocardial, Collagen, Heartworm

## Abstract

**Background:**

*Dirofilaria immitis* infection occurs in dogs and cats, both of which species are clinically affected by mature adult infections. Cats are uniquely affected by immature-adult infections with an inflammatory pulmonary disease called Heartworm-Associated Respiratory Disease (HARD). *D. immitis* infection causes pulmonary parenchymal and vascular pathology in the dog and cat. Dogs develop pulmonary hypertension and cor pulmonale, whereas the development of pulmonary hypertension is rare in the cat. *D. immitis* infection in the dog causes alteration of the right ventricular (RV) extracellular matrix, including a decrease in myocardial collagen. In this study, the RV myocardial changes of cats infected with adult and immature-adult *D. immitis* were assessed.

**Methods:**

The cardiopulmonary systems of six groups of SPF cats (*n* = 9-10 per group) were examined 8 or 18 months after infection with L3 *D. immitis*. Two groups were untreated and allowed to develop adult HW; two groups were treated with ivermectin starting 3 months post infection, thus allowing HARD but no mature adult heartworms; and two groups were treated with selamectin beginning 1 month post infection, preventing development of L5 or adult heartworms. A group of specific pathogen free (SPF) normal cats was utilized as a negative control (*n* = 12). Lung pathologic lesions were objectively assessed, and both RV and left ventricular (LV) weights were obtained to calculate an RV/LV ratio. Intramural RV myocardial collagen content was quantitatively assessed.

**Results:**

RV/LV weight ratios were not different between groups. Negative control cats had significantly greater RV collagen content than all other affected groups (*P* = 0.032). Analysis of the RV/LV ratios and collagen content revealed no significant relationship (*r* = 0.03, *P* = 0.723, respectively). Collagen content had a modest, but significant, negative correlation, however, with both pulmonary vascular pathology (*r* = −0.25, *P* = 0.032) as well as the total pulmonary parenchymal and vascular pathology (*r* = −0.26, *P* = 0.025).

**Conclusions:**

Cats infected with mature and immature *D. immitis* did not develop RV hypertrophy but did demonstrate loss of RV myocardial collagen content. The collagen loss was present at 8 and 18 months after infection in all infected cats. This loss of RV myocardial collagen was correlated with the severity of pulmonary parenchymal and vascular pathology.

## Background

*Dirofilaria immitis* infection occurs in both dogs and cats, with prevalence rates varying by geographical distribution [1, 2]. Prevalence rates in dogs have been reported as high as 80% in some areas, and prevalence rates of infections in cats are typically 5% to 20% of the canine infection rate [1, 3]. Clinical consequences of mature adult *D. immitis* infection is well-documented in dogs and cats, and cats also may develop significant clinical disease from immature-adult infections called Heartworm-Associated Respiratory Disease (HARD) [2, 4, 5].

Pathology associated with *D. immitis* infection in the cat includes significant pulmonary parenchymal pathology, in part due to significant pulmonary inflammation [5, 6]. In addition to pulmonary parenchymal pathology, decreased bronchiolar reactivity as well as pulmonary arterial pathology have been reported with *D. immitis* infection in the cat [5, 7, 8]. Adult heartworm disease in the dog causes similar degrees of vascular and pulmonary inflammation, with the development of pulmonary hypertension (PH) being relatively common [4, 9]. A clinical syndrome known as cor pulmonale occurs when PH develops to a degree severe enough to affect the right side of the heart. Typically, enlargement and hypertrophy of the right ventricle (RV) develop secondary to PH in dogs with cor pulmonale [10]. With severe changes to the RV, right atrial emptying is impeded, leading to right atrial enlargement and right-sided heart failure.

PH and right heart failure are rare in cats [2, 11]. Even with RV hypertrophy of dogs infected with *D. immitis*, the RV myocardium has extensive myocardial damage with a loss of extracellular matrix, including collagen [12, 13]. The RV myocardium has been evaluated in feline models of increased RV afterload (ie, pulmonary artery banding), and in contrast to the RV myocardium in dogs with heartworm disease, these models of increased RV afterload show an increased density and content of RV collagen [14, 15]. However, there are no reports of changes in the myocardium of cats infected with *D. immitis*. The purpose of this study was to evaluate the RV myocardium of cats infected with *D. immitis* and to correlate the observed myocardial disease with pulmonary parenchymal and vascular pathology. Our hypothesis was that the RV myocardial collagen content would be decreased in cats infected with *D. immitis*, similar to what has been reported in dogs infected with *D. immitis*. Additionally, we hypothesized that the decrease in RV myocardial content would correlate with the severity of pulmonary parenchymal and vascular pathology.

## Methods

### Experimental design

A total of 71 specific pathogen free (SPF), spayed, female, 6-month-old cats were divided into seven groups of 9 to 12 cats each. Groups were classified based on whether or not cats were infected with *D. immitis* larvae, whether and how cats were treated with macrocyclic lactones, and duration of the study (8 months or 18 months) (Table 1 **)**. One group of 12 cats was uninfected and untreated (UU), serving as a true negative control for 8 months, with no exposure to *D. immitis*. All remaining cats were infected with 100 *D. immitis* L3 (Missouri strain) by subcutaneous injection into the flank [16]. Two groups of 10 cats each were infected and untreated (to allow development of mature adult heartworms), and were observed for 8 months (IU-8) or 18 months (IU-18). Two groups of 10 cats each were infected and treated with selemectin (to prevent development of L5 larvae or HARD) and were observed for 8 months (IS-8) or 18 months (IS-18). Finally, two groups of cats (9 and 10 cats, respectively) were infected and treated 3 months later with ivermectin (to allow development of HARD and immature adult *D. immitis* but prevent development of mature adult *D. immitis*) and were observed for 8 months (IH-8) or 18 months (IH-18).

**Table 1:** Number of cats, infective L3 injected, treatments, and length of study for all cats (*n* = 71)

Group	Number of cats	Infective L3	Treatment	Length of study
UU	12	0	None	8 months
IU-8	10	100	None	8 months
IU-18	10	100	None	18 months
IS-8	10	100	Selamectin	8 months
IS-18	10	100	Selamectin	18 months
IH-8	9	100	Ivermectin	8 months
IH-18	10	100	Ivermectin	18 months

*Abbreviations:* IU-8 – infected, untreated group (8 months duration); IU-18 – infected, untreated group (18 months duration); IS-8 – infected, treated with selamectin 30 days postinfection group (8 months duration); IS-18 – infected, treated with selamectin 30 days postinfection group (18 months duration); IH-8 – infected, treated with ivermectin 72 day postinfection group (8 months duration); IH-18 infected, treated with ivermectin 72-day postinfection group (18 months duration): UU – uninfected, untreated group

Cats in the IS-8 and IS-18 groups were treated topically with selamectin (Revolution^®^, Zoetis) at the dosage based on body weight as indicated on the label 30 days postinfection and every 30 days for the remainder of the study in order to prevent the development of the L5 stage (see “Heartworm-Associated Respiratory Disease (HARD) induced by immature adult *D. immitis* in cats” and “The progression of Heartworm-Associated Respiratory Disease (HARD) in SPF cats 18 months after *D. immitis* infection” by A. Ray Dillon et al. in this proceedings) [17, 18]. Cats in the IH-8 and IH-18 groups were treated orally with ivermectin (Ivomec^®^, Merial), 150 mcg/kg per os on day 72 post infection and every 2 weeks for the remainder of the study to allow only immature adult heartworms to induce HARD lesions but no fully mature heartworms to develop [17, 19]. Cats were observed for a period of 8 or 18 months postinfection and monitored daily. Body weights were obtained at the beginning and end of the study. Physical examinations were performed weekly.

All groups of cats were housed as isolated groups in the indoor animal rooms of the Laboratory Animal Health Veterinary Research Building to prevent exposure to mosquitoes that could be carrying heartworm larvae. The study protocol was approved by the Auburn University Institutional Animal Care and Use Committee and conducted in an AAALAC-accredited facility in an environmentally isolated facility.

### Necropsy

At 8 or 18 months postinfection, cats were humanely euthanized under sedation, using pentobarbital sodium and phenytoin sodium solution (Euthasol^®^, Virbac Animal Health) via 1 mL/10 lb. intraperitoneal injection. Complete necropsies were performed with collection of lung, heart, brain, kidney, and liver for histopathologic studies. The number of adult heartworms observed at necropsy was recorded. Lung lobes were fixed by perfusion with 10% formalin via the bronchi to a pressure of 14 cm H_2_O.

### Lung histopathology

Multiple sections of the fixed-perfused right caudal lung lobe were stained with hematoxylin and eosin. Lung pathology was objectively assessed by observers who were blinded to group designation and were trained for this assessment of lung histopathology by boarded veterinary pathologists. Specific anatomic areas evaluated for pathology included the bronchi, bronchioles, alveoli, alveolus (smooth muscle), pulmonary arteries, and pulmonary arterioles. All subjects had pathology severity scores (0–3 range) assigned for each anatomic area evaluated within the lung [5]. Pathology severity scores were then combined as two different combination values: the total lung pathology (TLP) score, which included pathology scores combined from all areas evaluated, and the pulmonary artery and arteriole (PAA) score, which included the combined vascular pathology scores from the pulmonary arteries and arterioles.

### Right ventricular collection and histopathology

At the time of necropsy, total heart weights were obtained. The atria and great vessels were dissected away from the ventricles by one author (ARD), and weights were obtained for the left ventricle (LV) and RV separately, as the RV free wall was dissected away from the interventricular septum (thus leaving LV and interventricular septum together). Roughly 1 × 3 cm sections were removed by one author (RLW) from the right ventricular free wall, opposite and slightly ventral to the crista supraventricularis. Myocardial sections were embedded in paraffin, and 4 μm thick samples stained with Picrosirius Red (Sigma-Aldrich) and photographed for assessment of collagen. Collagen content of these RV samples was quantified using a commercially available software program designed for histologic analysis (Visiopharm). This software program has the ability to quantify aspects of many different tissue stains, with user input training the software to identify the various color components of these stains correctly [19, 20]. Within each RV sample, epicardial and periarterial collagen were excluded from analysis, such that only myocardial collagen content (collagen to non-collagen ratio) was assessed.

### Statistical analysis

All statistical analyses were performed with commercially available statistical software (JMP^®^ Pro 11.1.1, SAS Institute Inc). Data were assessed for normality via the Shapiro-Wilk test and expressed as mean +/− SEM or median and range as appropriate. Comparisons between groups were performed by ANOVA or Kruskal-Wallis tests. Analysis of correlation was assessed by Spearman’s rank correlation and simple linear regression. The level of significance was considered as *P* ≤ 0.05.

## Results

### RV analysis

**Fig. 1:**
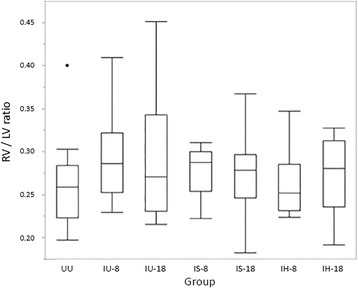
Box and whisker plots of right ventricle (RV) to left ventricle (LV) weight ratios by treatment group. *Abbreviations:* IU-8 – infected, untreated group (8 months duration); IU-18 –infected, untreated group (18 months duration); IS-8 – infected, treated with selamectin (8 months duration) group; IS-18 – infected, treated with selamectin (18 months duration) group; IH-8 – infected, treated with ivermectin (8 months duration) group; IH-18 – infected, treated with ivermectin (18 months duration) group; UU – uninfected, untreated group

**Fig. 2:**
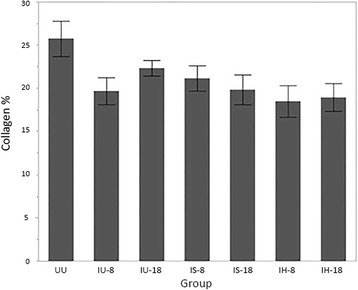
Mean RV collagen content percentage by treatment groups. Error bars = SEM. *Abbreviations:* IU-8 – infected, untreated group (8 months duration); IU-18 –I nfected, untreated group (18 months duration); IS-8 – infected, treated with selamectin (8 months duration) group; IS-18 – infected, treated with selamectin (18 months duration) group; IH-8 – infected, treated with ivermectin (8 months duration) group; IH-18 – infected, treated with ivermectin (18 months duration) group; UU – uninfected, untreated group

**Fig. 3:**
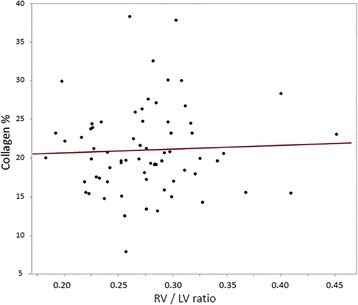
Simple linear regression plot of RV myocardial collagen content percentage by the ratio of right ventricle (RV) to left ventricle (LV) weight

The RV/LV ratio for normal cats (UU group) was a median of 0.26 with a range of 0.20 to 0.40. There were no differences in RV/LV ratio between groups (*P* = 0.632; Fig. 1). Histopathologic analysis revealed an overall collagen percentage of the right ventricle in the UU group to be 25.7% +/− 7.1%. Significant differences in RV collagen percentage were observed between groups (*P* = 0.032), with the UU group having the highest RV collagen percentage (Fig. 2). Post hoc analysis revealed the UU group RV collagen percentage was significantly greater than that of the IH-8 group (*P* = 0.049). Comparison of the RV/LV weight ratio with RV collagen percentage for all cats revealed a poor correlation (*r* = 0.03) that was statistically insignificant (*P* = 0.723; Fig. 3).

### Lung histopathology

**Fig. 4:**
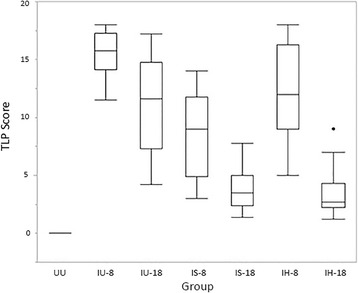
Box and whisker plots of total lung pathology (TLP) severity scores by groups. *Abbreviations:* IU-8 – infected, untreated group (8 months duration); IU-18 – infected, untreated group (18 months duration); IS-8 – infected, treated with selamectin (8 months duration) group; IS-18 – infected, treated with selamectin (18 months duration) group; IH-8 – infected, treated with ivermectin (8 months duration) group; IH-18 – infected, treated with ivermectin (18 months duration) group; UU – uninfected, untreated group

Adult heartworms were present in IU-8 (10/10) and IU-18 (9/10) but in none of IH-8, IH-18, IS-8, or IS-18 groups. The overall TLP score for all cats in the UU group was 0. Cats infected with L3 (ie, all cats not in UU group) had a TLP median of 9 with a range of 1.2 to18, with 18 being the maximum score (Fig. 4). Significant differences in TLP score were observed between groups (*P* = <0.001). The three groups with the highest TLP scores were the IU-8 group (15.75, range 11.5-18), the IH-8 group (12, range 5-18), and the IU-18 group (11.6, range 4.2-17.2). TLP scores from cats in the IS-8 group were significantly greater than TLP scores from cats in the IS-18 group (*P* = 0.002). TLP scores from cats in the IH-8 group were significantly greater than TLP scores from cats in the IH-18 group (*P* = <0.001). Individual cat TLP scores varied widely within each group.

**Fig. 5:**
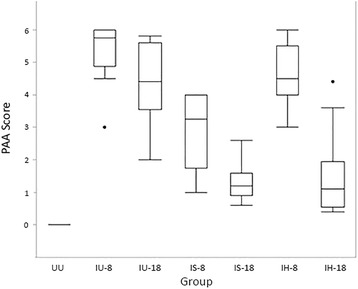
Box and whisker plots of combined pulmonary artery and arteriole (PAA) pathology severity scores by groups. *Abbreviations:* IU-8 – infected, untreated group (8 months duration); IU-18 –infected, untreated group (18 months duration); IS-8 – infected, treated with selamectin (8 months duration) group; IS-18 – infected, treated with selamectin (18 months duration) group; IH-8 – infected, treated with ivermectin (8 months duration) group; IH-18 – infected, treated with ivermectin (18 months duration) group; UU – uninfected, untreated group

The PAA score for all normal cats was 0. Cats infected with L3 (ie, all cats not in UU group) had a PAA median of 3.6 with a range of 0.4-6 (Fig. 5). There were significant differences between groups (*P* = <0.001). The three groups with the highest PAA scores were the IU-8 group (5.75, range 3-6), the IH-8 group (4.5, range 3-6), and the IU-18 group (4.4, range 2-5.8). PAA scores from cats in the IS-8 group were significantly greater than PAA scores from cats in the IS-18 group (*P* = 0.001). PAA scores from cats in the IH-8 group were significantly greater than PAA scores from cats in the IH-18 group (*P* = <0.001).

### Relationship of RV collagen to lung histopathology

**Fig. 6:**
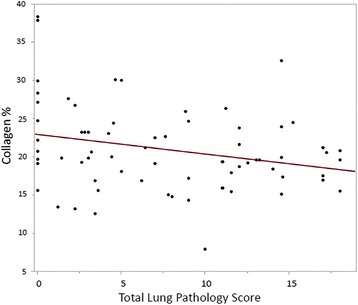
Simple linear regression plot of RV myocardial collagen percent by total lung pathology severity scores

**Fig. 7:**
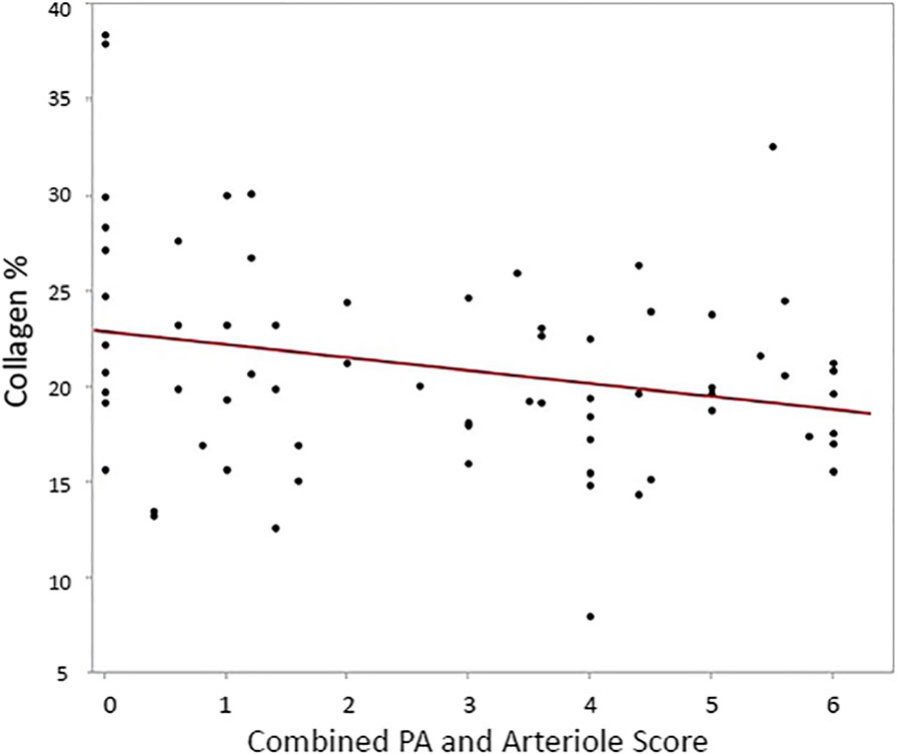
Simple linear regression plot of RV myocardial collagen percent by combined pulmonary arterial and arteriolar severity scores

The TLP scores of all cats and RV collagen percentage values were compared, and the RV collagen percentage decreased as the TLP score increased (Fig. 6). This relationship had a moderate negative correlation (*r* = −0.26) that was statistically significant (*P* = 0.032). A similar result was observed when comparing the PAA scores of all cats with RV collagen percentages (Fig. 7). The RV collagen percentage decreased as the PAA scores worsened, with a moderate negative correlation (*r* = −0.25) that was statistically significant (*P* = 0.025).

## Discussion

This study is the first to describe changes in the RV of cats infected with L3/L4 stages, immature adults in pulmonary arteries and mature adult heartworms. Based on the RV weights and RV/LV ratio in infected cats (Fig. 1), there was no evidence of right ventricular hypertrophy which is the hallmark of pressure overload cardiac compensation. This may indicate that cats infected with *D. immitis* do not experience an increased pulmonary arterial pressure overload. In experimental models of banding of the pulmonary artery, the resultant increased afterload and vascular resistance induces RV concentric hypertrophy and increased right ventricular myocardial collagen in multiple species including the cat [15, 21, 22]. Compared with healthy, uninfected cats, those infected with *D. immitis* in this study developed a decrease in RV myocardial collagen and no evidence of RV hypertrophy. This decrease in myocardial collagen was significantly correlated with the severity of pulmonary parenchymal and arterial damage. RV collagen content decreased as TLP and PAA were more severe, which may suggest that as cats were more affected by their heartworm infection, their RV were increasingly affected. The observed decrease in RV collagen even in the two groups with only L3/L4 stages of *D. immitis* (selamectin treated) is consistent with circulating factors altering the RV collagen.

The decreased collagen observed in the cats in this study contradicts feline models of increased pulmonary afterload associated with increased RV collagen [14, 15]. Right-sided heart failure with cor pulmonale is an uncommon clinical presentation even in cats with heartworm disease and severe lung parenchymal disease [4, 23]. The present study involved cats with extensive pulmonary parenchymal and arterial damage, some with long-term PA luminal occlusion, which did not develop clinical evidence of increased pulmonary afterload (ie, pulmonary arterial hypertension) or RV hypertrophy. Pulmonary hypertension has been reported in cats with heartworm disease but not as commonly as it is observed in dogs with heartworm disease [2].

As a decrease in RV collagen content has been reported in dogs and in the cats reported herein, heartworm infection may be associated with a decrease in RV collagen that is independent of the presence of pulmonary hypertension. Dogs experimentally infected with *Angiostrongylus vasorum* develop significant pulmonary pathology, including pulmonary arterial thrombosis [24]*.* However, development of PH in these dogs is rare and believed to be in part due to the development of collateral circulation [25]. Despite the fact that dogs with heartworm disease do develop PH, it is possible that cats infected with heartworm disease may develop pulmonary collateral circulation, which prevents the development of PH.

Heartworm disease in dogs is commonly associated with pulmonary arterial hypertension, but one study reported a decrease in RV collagen content of 24 infected dogs with moderate to severe heartworm infections [12]. The presence or absence of pulmonary hypertension was unknown in that study. Indirect interactions between the host environment and heartworm parasite have been described. Antigens excreted and/or secreted from heartworms were found to alter the fibrinolytic system in vivo and vascular endothelial cells in vitro to promote clot lysis [26]. The authors of that study speculated that promoting clot lysis constituted a survival benefit for *D. immitis*. Another study supported the concept that heartworm-induced alteration of the fibrinolytic system participated in the induction of pulmonary endarteritis [27].

Based on in vitro evidence, heartworm antigens promote extracellular matrix degradation of pulmonary arteries [28]. This response may be the direct interaction of an excretory or secretory antigen with the feline RV. In the present study, although the selamectin-treated cats (treated 30 days post infection) developed no cardiac stages of heartworms and only precardiac stages of L3 and L4, the evidence of collagen loss in the RV of these cats is consistent with circulating soluble HW products inducing the RV changes. Further investigation into this phenomenon is warranted. The possibility of the collagen loss being permanent is suggested by the decrease in collagen that persists 18 months after infection even in the infected selamectin-treated group.

This study has limitations. Invasive pulmonary arterial pressure measurements were not obtained, and therefore the presence or absence of pulmonary hypertension could not be determined. Thus, a correlation between pulmonary pathology and measured pulmonary hypertension could not be determined. Additionally, echocardiography was not obtained in these cats, therefore the relationship between the loss of RV myocardial collagen content and in gross changes, such as RV dilation, is unknown. However, a study utilizing the same experimental design for 8-month groups did have sequential echocardiography and CT scans performed, and no pulmonary hypertension was noted in cats with adult heartworms [5]. All myocardial samples were immediately stored in formalin at the time of euthanasia, and this method of fixation precludes the use of many special stains. Therefore, the results of this study indicate a loss of RV myocardial collagen without a decrease in RV weights, but it cannot be determined if there were increases in other components of the myocardial interstitium in response to heartworm infection.

## Conclusions

Cats infected with immature stages of *D. immitis* and mature adult *D. immitis* develop a loss of RV myocardial collagen content but no change in RV mass at 8 and 18 months after infection. This loss of RV myocardial collagen is correlated with the severity of pulmonary parenchymal and arterial pathology and persists even many months after resolution of an immature *D. immitis* infection.

## Abbreviations

HARD: Heartworm-Associated Respiratory Disease
IH-18: Infected, treated with ivermectin group (18-month study duration)
IH-8: Infected, treated with ivermectin group (8-month study duration)
IS-18: Infected, treated with selamectin group (18-month study duration)
IS-8: Infected, treated with selamectin group (8-month study duration)
IU-18: Infected, untreated group (18-month study duration)
IU-8: Infected, untreated group (8-month study duration)
LV: Left ventricle
PAA: Pulmonary artery and arteriole
PH: Pulmonary hypertension
RV: Right ventricle
TLP: Total lung pathology
UU: Uninfected, untreated group
